# Supplementary material for: Detection of Nail Oncometabolite SAICAR in Oral Cancer Patients and Its Molecular Interactions with PKM2 Enzyme
Source: Int J Environ Res Public Health. 2021 Oct 26;18(21):11225. doi: 10.3390/ijerph182111225 (PMC8583651; doi:10.3390/ijerph182111225)
Supplement: Supplementary file 1 [file ijerph-18-11225-s001.zip › ijerph-1370786-supplementary.pdf]

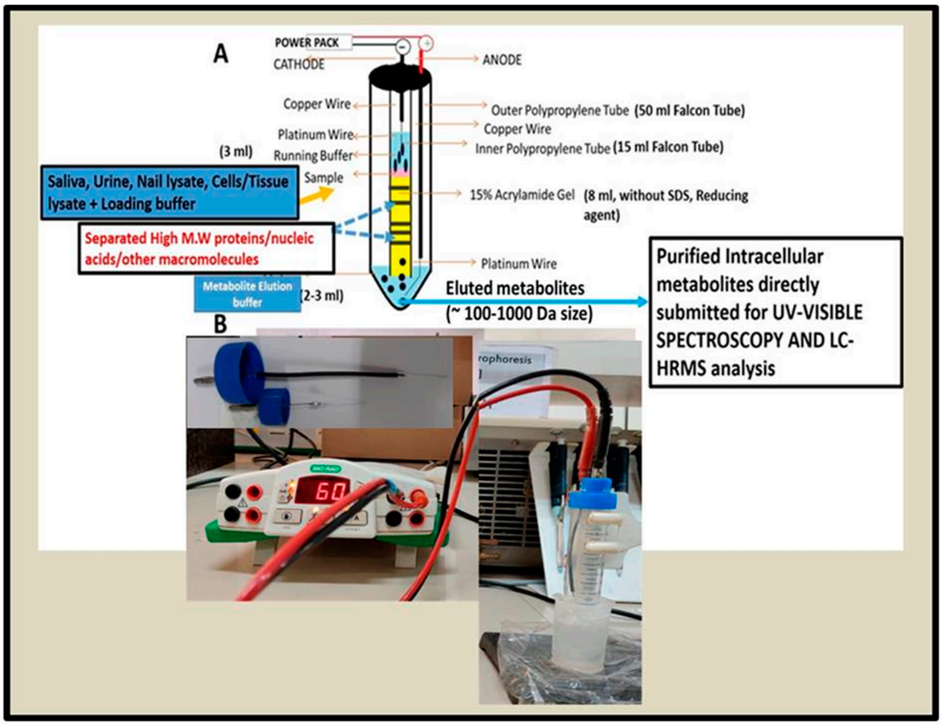

Figure S1. A model on a novel vertical tube gel electrophoresis (VTGE) system. (**A**) Flow diagram of VTGE system. (**B**) A running model on VTGE system.
